# A prospective Swedish study on body size, body composition, diabetes, and prostate cancer risk

**DOI:** 10.1038/sj.bjc.6605077

**Published:** 2009-05-12

**Authors:** P Wallström, A Bjartell, B Gullberg, H Olsson, E Wirfält

**Affiliations:** 1 Nutrition Epidemiology Research Group, Department of Clinical Sciences, Lund University, Malmö, Sweden; 2 Division of Urological Cancers, Department of Clinical Sciences, Lund University, Malmö, Sweden; 3 Cancer Epidemiology Unit, Section V, Department of Clinical Sciences, Lund University, Lund, Sweden

**Keywords:** prostatic neoplasms, cohort studies, body composition, body fat distribution, body height (or diabetes mellitus)

## Abstract

Obesity may be associated with increased risk of prostate cancer (PCa). According to one hypothesis, obesity could lower the risk of non-aggressive tumours, while simultaneously increasing the risk of aggressive cancer. Furthermore, central adiposity may be independently associated with PCa risk; it is also associated with diabetes, which itself may influence risk of PCa. We studied the associations between height, body composition, and fat distribution, diabetes prevalence and risk of total, aggressive, and non-aggressive PCa in 10 564 initially cancer-free men (aged 45–73 years) of the population-based Malmö Diet and Cancer cohort. Anthropometric and body composition measurements, including bioelectrical impedance for estimation of fat mass, were performed by study nurses. Diabetes prevalence was self-reported. Cancer cases and clinical characteristics were ascertained through national and regional registry data. Dietary and other background data were obtained through a modified diet history method and an extensive questionnaire. During a mean follow-up of 11.0 years, 817 incidental PCa cases were diagnosed. Of these, 281 were classified as aggressive. There were 202 cases occurring before 65 years of age. Height was positively associated with total and non-aggressive PCa risk. Waist–hip ratio (WHR), a measure of central adiposity, was positively associated with PCa before age 65, and less strongly, with total PCa. This association was independent of body mass index (BMI) and other potential confounders. General adiposity, expressed as BMI or body fat percentage, and prevalent diabetes were not associated with PCa risk. In this study, WHR and body height were stronger PCa predictors than general adiposity.

Obesity has long been associated with increased risk of prostate cancer (PCa), although studies have been inconsistent (MacInnis and English, 2006). Risk has been suggested to differ according to tumour grade: obesity lowering the risk of indolent, less aggressive tumours, whereas increasing that of aggressive cancer (Freedland *et al*, 2006), a view supported by a meta-analysis (MacInnis and English, 2006), and, to some extent, by other work (Gong *et al*, 2006; Littman *et al*, 2007; Rodriguez *et al*, 2007; Wright *et al*, 2007). Most studies have used body mass index (BMI) as a marker of general obesity, but it has certain limitations, particularly in men, because it does not differentiate muscle from fat mass (Willett, 1998). One solution might be to calculate fat mass and fat-free mass from bioelectrical impedance (BIA) measurements (Anonymous, 1996; MacInnis *et al*, 2003).

There is less support for the hypothesis that central adiposity (measured as waist circumference or waist—hip ratio, WHR) is a risk marker for PCa (MacInnis and English, 2006). Only a few prospective studies have examined this association (Giovannucci *et al*, 1997; Lee *et al*, 2001; MacInnis *et al*, 2003; Hubbard *et al*, 2004). Furthermore, central adiposity is a clinically established risk factor for non-insulin dependent diabetes mellitus (NIDDM) (Vazquez *et al*, 2007; Gastaldelli, 2008), and NIDDM has been associated with lower risk of PCa (Bonovas *et al*, 2004), both low grade and high grade (Gong *et al*, 2006). High insulin levels, as in insulin resistance, which often precedes NIDDM, was recently associated with lower risk of non-aggressive PCa (Stocks *et al*, 2007), although there was also a non-significant positive association with aggressive cancer.

Tallness has also been associated with PCa risk (Engeland *et al*, 2003; MacInnis and English, 2006); perhaps reflecting an influence of nutritional factors.

We investigated total, aggressive, and non-aggressive PCa in relation to current obesity, body composition, height, and prevalence of diabetes mellitus in a population-based cohort, including a sub-cohort of younger men.

## Materials and methods

The background population of the Malmö Diet and Cancer (MDC) study, in Sweden's third largest city (Berglund *et al*, 1993), consists of all men born in 1923–1945 and all women born in 1923–1950 who were living in Malmö during the screening period 1991–1996 (*n*=74 138). This population was identified through national population registries, the final cohort consisting of 28 098 individuals (participation rate 40.8%). Participants were recruited through advertisements in local media and through invitation by mail. The only exclusion criteria were inadequate Swedish language skills and mental incapacity (Manjer *et al*, 2001, 2002); the Ethics Committee at Lund University approved the design of the MDC study (LU 51–90).

Waist circumference was measured midway between the lowest rib margin and iliac crest; hip circumference horizontally at the level of the greatest lateral extension of the hips. Bioelectrical impedance was used for estimating body composition according to manufacturer's procedures (BIA 103, RJL-systems, Detroit, MI, USA; single-frequency analyser). An algorithm (Sun *et al*, 2003) was used to estimate body fat (BF) from impedance. The results were highly correlated to results obtained by another algorithm, developed in a similar population (Heitmann, 1990). Estimated BF was used to calculate BF%.

A structured multiple-choice questionnaire was used in the MDC study to collect information on sociodemographic factors, smoking status, alcohol habits, health status, use of pharmaceutical drugs, and several other factors. The agreement between baseline questionnaire and its repeat after 3 weeks was high for most variables (*κ* values >0.75) (Manjer *et al*, 2002). The diabetes items read: ‘Have you ever been treated for diabetes? Since what year?’ Persons who reported using oral anti-diabetic drugs were also classified as diabetics.

The diet assessment, reported earlier (Wirfält *et al*, 2002), combines quantitative and semi-quantitative approaches to the entire diet, including cooking methods (Callmer *et al*, 1993). It consists of two parts: a ‘menu book’ for description of cooked meals and registration of cold (including juices and alcoholic) beverages and dietary supplements during seven consecutive days; and a 168-item food questionnaire on regularly consumed foods during the past year. Data on validity (Elmståhl *et al*, 1996b; Riboli *et al*, 1997) and reproducibility (Elmståhl *et al*, 1996a) have been reported.

Cancer cases were ascertained by record linkage with the National Cancer Register. Cancer cases from the year 2005, additional data on tumour stage and grade, pre-diagnostic serum prostate-specific antigen (PSA) value, and reason for diagnosis (symptoms, health examination, or other) were obtained from the National Prostate Cancer Register (NPCR) (South Region). In the South Region, to which Malmö belongs, registration was started in 1996 and is at least 95% complete. For cases diagnosed in 1991–1995, the same data were manually extracted from medical records using standard routines. The National Cancer Register is known to be at least 98% complete. A validation of the NPCR data from another region showed high validity for all variables, including one used to classify non-aggressive and aggressive tumours (Sandblom *et al*, 2003).

Of the 11 063 men in the MDC cohort, 485 were already diagnosed with cancer (excluding basal cell carcinomas) at study entry, and were therefore excluded; 14 others were excluded because of PCa diagnosis at autopsy, leaving 10 564 men for analysis. They were followed until date of death, date of PCa diagnosis, or 31 December 2005, whichever came first. No participants were lost to follow-up of vital status. Among them, 817 incident cases of PCa (‘total PCa’; ICD-9 code 185) occurred between baseline examination and end of follow-up. The average follow-up time in participants free of PCa at death (*n*=1321) or at the end of follow-up (*n*=8426) was 11.0 years.

An aggressive case (‘aggressive Pca’) was defined as a tumour with a clinical T stage of 3 or higher *or* tumour-positive lymph nodes (N1) *or* one or more distant metastases (M1) *or* a Gleason score of 8 or higher *or* a pre-treatment serum PSA value of at least 50 ng ml^−1^ (Stocks *et al*, 2007); tumours were also classified as aggressive (total 281) if the WHO grade was 3, and Gleason score was unavailable (*n*=6). In cases in which at least two of T stadium, Gleason score, or PSA serum value were reported, and if none of these factors indicated an aggressive tumour, the tumour was classified as non-aggressive (*n*=530). Staging data were unavailable or insufficient for 6 of the incident cases that with 530 cases of non-aggressive cancers were excluded from analyses comparing men with aggressive tumours with others. Details on stage and grade have been reported earlier (Wallström *et al*, 2007).

For PCa in younger people, we repeated all analyses in a sub-cohort, the <65 sub-cohort, the end of follow-up being either at date of death, date of PCa diagnosis, 31 December 2005, or the man's 65th birthday, whichever came first. This younger sub-cohort consisted of 8194 men, among whom 202 incident PCa cases (54 aggressive) occurred. Mean follow-up time for non-cases was 7.7 years.

We examined the associations between total, aggressive, and non-aggressive PCa incidence and height, waist circumference, waist-hip ratio (WHR), BMI, BF%, and prevalence of diabetes. All continuous variables were divided into quintile groups. The hazard ratios (HR) of each distribution quintile (compared with the lowest), and trends across the quintiles were assessed with Cox proportional hazards regression with adjustment for age at baseline. The time variable was number of days of follow-up after baseline. Additionally, in a separate Cox analysis, BMI was divided into pre-defined categories (World Health Organization, 2000) with tests for overall differences between categories. We also performed analyses of waist circumference and WHR with adjustment for BMI, to assess risk associated with abdominal, irrespective of general, adiposity. All analyses were age-adjusted and were also performed in the <65 sub-cohort (202 cases). We then repeated the above analyses with adjustment for a number of potential confounders, selected from a survey of the current scientific literature: co-habitation status, socioeconomic index, alcohol habits, BF%, smoking history, birth country (Sweden/other), total calcium intake, dietary intake of eicosapentaenoic acid and docosahexaenoic acid, consumption of fruits, vegetables, and red meat. The dietary variables were adjusted for total energy intake (residual method) (Willett and Stampfer, 1998).

To further evaluate the consistency of our results, we repeated all analyses (including subgroups) in three separate sessions. In the first, we excluded asymptomatic cases (478 cases remaining in the total group). In the second, we excluded persons not born in Sweden (748 cases), and in the third, we excluded cases occurring within two years of the beginning of follow-up (691 remaining cases).

## Results

Height was associated with increased risk of total PCa, although the effect was limited to the highest quintile (range 182–203 cm), and was most evident among non-aggressive tumours (Table 1). No other measures of body composition or fat distribution were significantly associated with PCa risk, although being underweight (BMI <18.5) was associated with higher risks than normal weight across all PCa categories except the non-aggressive category; an association with BMI-adjusted WHR was suggested. Neither prevalent diabetes was significantly associated with PCa risk nor height in the <65 sub-cohort (Table 2, age-adjusted analyses), there was a weak, positive association with WHR. After adjustment for BMI, the association was slightly stronger (HR per quintile 1.14, 95% CI 1.03–1.26). Similarly, waist circumference (adjusted for BMI) was positively associated with risk in the <65 sub-cohort.

After adjustment for a number of possible confounders, the increased risk of total PCa associated with greater height was weakened (*P* for trend=0.08, Table 3). However, the highest quintile was still significantly different from the lowest (*P*=0.018). The associations between WHR and PCa, both in total and in the <65 sub-cohort, were somewhat stronger after multivariate adjustment (Tables 2 and 3). The associations with being underweight were also slightly stronger after adjustment (Table 2); otherwise, the results were similar. Inspection of the data revealed no suggestion of any significant interactions between any body measure and aggressive *vs* non-aggressive tumours.

The association between high stature (tallest quintile) and risk of total PCa was a robust finding in most models (exclusion of persons born outside of Sweden, asymptomatic cases, and cases occurring within 2 year of screening, respectively). This was true of the waist/WHR models, both univariate and multivariate. However, the association between being underweight according to BMI classification and increased risk of total PCa was greatly weakened after removal of cases occurring within the first 2 years or asymptomatic cases. Apart from these, the sensitivity analyses yielded no consistent results.

## Discussion

This study showed a weak association between tallness and risk of total PCa, also shown (but not significantly) by aggressive PCa or PCa in the <65 sub-cohort. The role of height in PCa, if any, is controversial (Gunnell *et al*, 2001). A similar finding was reported in the largest study to date, examining 950 000 Norwegian men, in which the tallest men (⩾190 cm) had an RR of 1.72 (95% CI 1.46–2.04) compared with the shortest (Engeland *et al*, 2003). Although an RR greater than 1.00 for the tallest men has been reported several times, this was not usually significant at a 0.05 level (Gunnell *et al*, 2001). A recent study from the EPIC cohort found no association between height and risk, neither for aggressive nor for non-aggressive tumours (Pischon *et al*, 2008). Furthermore, height was recently associated with increased PCa mortality (Giovannucci *et al*, 2007).

To our knowledge, height in itself has not been regarded as a causal factor for PCa, but rather associations have been attributed to genetic, hormonal, and nutritional factors related to growth until adulthood, both before and after birth (Gunnell *et al*, 2001). As there is evidence of a positive association between body height and socioeconomic class, perhaps reflecting factors such as a greater abundance of food during growth or lower incidence of infections, one could imagine socioeconomic class confounding the association between height and PCa (Batty *et al*, 2006). In this study, however, this was only marginally affected by adjustment for socioeconomic index and other potential confounders.

Few studies have reported associations between waist/WHR and PCa; a recent meta-analysis judged the evidence to be weak (MacInnis and English, 2006). In our study, WHR (and, to a smaller extent, waist) was associated with PCa, particularly before age 65 and after adjustment for BMI. Detection bias is possible: persons with higher WHR may be more likely to have other health problems and therefore visit a doctor, which could increase the chance of diagnosis. However, the sensitivity analysis showed, if anything, stronger associations when asymptomatic cases were excluded (data not shown). Although usually highly correlated with BMI, there are data to suggest that waist circumference and/or WHR may predict health problems independently of BMI, as in diabetes mellitus (Gastaldelli, 2008) and CVD (Yusuf *et al*, 2005). Furthermore, there is accumulating evidence for a role of waist circumference (Visscher *et al*, 2001; Koster *et al*, 2008) and WHR (Lahmann *et al*, 2002; Price *et al*, 2006) in predicting mortality independently of BMI in men. A *post hoc* analysis of total PCa in the present cohort shows that the increased PCa risk associated with large waist circumference seemed to be greatest at low BMI (data not shown), a finding that was not explained by prevalent asymptomatic cancer.

It is not clear why the association between WHR and PCa risk was most pronounced in the <65 sub-cohort, or why height was not associated with aggressive PCa. Chance findings can never be entirely ruled out; although the number of factors examined in this study was limited and well defined, the actual number of statistical tests was large. On the other hand, our findings might reflect that unmeasured factors associated with high WHR/waist circumference become relatively less important to PCa development as age increases. Similarly, height may be a marker of biological processes leading to less aggressive PCa.

General adiposity was not associated with PCa in this study. The HR estimates for BF% were slightly different from those for BMI, but not convincingly so. As the use of BF% as a marker of general adiposity has recently been criticised (Cole *et al*, 2008), we repeated our main analyses with BF% replaced by BF mass adjusted for lean body mass, but with broadly similar results (data not shown). One obvious explanation is that there may be no effect or a very small effect of general adiposity on PCa risk (MacInnis and English, 2006; Renehan *et al*, 2008), or that BF% is not different enough from BMI to capture any differences in this respect.

Underweight (BMI <18.5) was associated with all categories of PCa. This may represent weight loss owing to pre-clinical cancer, particularly since the associations disappeared after exclusion of cases occurring within the first 2 years after screening.

Diabetes was not significantly associated with incidence of PCa in this study. Several studies linking NIDDM and/or insulin resistance with lower risk of PCa have been published in recent years (Bonovas *et al*, 2004; Gong *et al*, 2006; Stocks *et al*, 2007). As noted above, we used self-reported data on diabetes prevalence. However, because we also defined diabetics by their oral anti-diabetic drug use, only diabetics without oral therapy (or who failed to record it) and who did not answer the diabetes item correctly should be misclassified. This predominantly NIDDM group may be mixed with type 1 diabetes, because the questionnaire did not differ between diabetes types. However, few persons reported age at start of diabetes treatment <36 years (*n*=30, 12% of cases). Finally, it should be noted that our power to detect an association was rather low, because of the relatively small number of diabetics.

Many researchers use a Gleason score of 7+ instead of 8+ for defining more aggressive tumours. Changing our definition of aggressive to include Gleason 7 meant resulted in another 99 cases among the aggressive, with unchanged null results and weaker positive results (data not shown). This could be interpreted as an effect of diluting the aggressive group of cases by adding a large number of less malignant tumours.

In this population-based cohort study, we noted positive associations between risk of PCa and adult height, WHR, and waist circumference. No other measures of obesity or body size, or prevalent diabetes were significantly associated with PCa risk. There were no suggestions of significant interactions between measures of obesity and degree of aggressiveness of the tumours.

## Conflict of interest

The authors declare no conflict of interest.

## Figures and Tables

**Table 1:** Age-adjusted hazard ratios (with lower and upper limits of 95% confidence intervals) for measures of height, obesity, and diabetes prevalence and total, aggressive, and non-aggressive cases of prostate cancer^a^

				**Incident prostate cancer, entire cohort (*n*=10 564)^b^**											
			**Non-cases (*n*=9747)**	**All cases (*n*=817)**				**Aggressive cases (*n*=281)**				**Non-aggressive cases (*n*=530)**			
	**Median**	**Range**	***N***	***N***	**HR**	**Lower**	**Upper**	***N***	**HR**	**Lower**	**Upper**	***N***	**HR**	**Lower**	**Upper**
*Height (cm)*															
Quintile 1	168	⩽170	1798	144	1.00			50	1.00			93	1.00		
Quintile 2	173	171–174	2011	182	1.20	0.97	1.49	71	1.40	0.97	2.01	109	1.10	0.84	1.46
Quintile 3	177	175–178	2263	182	1.11	0.89	1.38	65	1.20	0.83	1.74	115	1.06	0.81	1.39
Quintile 4	180	179–181	1536	116	1.09	0.85	1.39	36	1.06	0.69	1.63	80	1.12	0.83	1.51
Quintile 5	184	⩾182	2123	193	1.40	1.13	1.74	59	1.38	0.95	2.02	133	1.42	1.09	1.86
					*0.016*				*0.37*				*0.014*		
*BMI*															
Underweight	n/a	⩽18.49	48	8	2.09	1.03	4.22	4	2.88	1.06	7.82	4	0.60	0.22	1.61
Normal	n/a	18.50–24.99	3627	287	1.00			102	1.00			183	1.00		
Overweight	n/a	25.00–29.99	4803	417	1.04	0.90	1.21	140	0.97	0.75	1.25	274	0.65	0.24	1.75
Obese	n/a	⩾30.00	1253	105	1.04	0.84	1.30	35	0.95	0.65	1.40	69	0.65	0.24	1.78
*Non-linear P*					*0.24* c				*0.20* d				*0.65* e		
															
*BMI*															
Quintile 1	22.2	⩽23.4	1949	158	1.00			61	1.00			96	1.00		
Quintile 2	24.4	23.5–25.2	1957	164	0.97	0.78	1.20	53	0.80	0.55	1.15	110	1.07	0.82	1.41
Quintile 3	26.0	25.2–26.8	1928	168	0.98	0.79	1.22	59	0.87	0.61	1.25	107	1.04	0.79	1.38
Quintile 4	27.7	26.8–28.7	1940	175	1.03	0.83	1.28	57	0.85	0.59	1.22	117	1.15	0.88	1.51
Quintile 5	30.6	⩾28.7	1957	152	0.91	0.73	1.14	51	0.77	0.53	1.12	100	1.00	0.76	1.33
					*0.67*				*0.27*				*0.80*		
*Body fat %*															
Quintile 1	15.7	⩽18.3	1952	146	1.00			53	1.00			93	1.00		
Quintile 2	19.9	18.3–21.3	1915	184	1.23	0.99	1.52	72	1.33	0.93	1.89	108	1.14	0.86	1.50
Quintile 3	22.6	21.3–23.8	1941	157	1.04	0.83	1.31	54	0.99	0.68	1.44	102	1.06	0.80	1.41
Quintile 4	25.1	23.8–26.6	1933	166	1.07	0.86	1.33	46	0.81	0.55	1.21	120	1.22	0.93	1.60
Quintile 5	28.8	⩾26.6	1937	161	1.08	0.86	1.35	55	1.01	0.69	1.48	105	1.11	0.84	1.47
					*0.98*				*0.25*				*0.37*		
*Waist (cm)*															
Quintile 1	82	⩽85	1960	152	1.00			58	1.00			94	1.00		
Quintile 2	88	86–90	1947	146	0.92	0.74	1.16	49	0.81	0.55	1.19	96	0.99	0.74	1.31
Quintile 3	93	91–95	2013	183	1.10	0.89	1.37	60	0.94	0.65	1.35	120	1.18	0.90	1.54
Quintile 4	98	96–101	1893	171	1.09	0.87	1.35	54	0.89	0.61	1.29	116	1.20	0.92	1.58
Quintile 5	107	⩾102	1912	165	1.04	0.83	1.30	60	0.96	0.67	1.38	104	1.08	0.82	1.43
					*0.35*				*0.99*				*0.26*		
*WHR*															
Quintile 1	0.87	⩽0.90	1945	161	1.00			58	1.00			96	1.00		
Quintile 2	0.91	0.90–0.93	1949	161	1.02	0.82	1.27	53	0.96	0.66	1.39	110	1.04	0.79	1.36
Quintile 3	0.94	0.93–0.96	1958	152	0.97	0.78	1.21	56	1.01	0.70	1.46	107	0.95	0.72	1.25
Quintile 4	0.97	0.96–0.99	1913	187	1.21	0.98	1.50	67	1.23	0.87	1.75	117	1.20	0.92	1.56
Quintile 5	1.02	⩾0.99	1959	156	1.08	0.87	1.35	47	0.94	0.64	1.39	100	1.13	0.86	1.48
					*0.17*				*0.67*				*0.21*		
*Diabetes?*															
No	n/a	n/a	9336	790	1.00			273	1.00			511	1.00		
Yes	n/a	n/a	411	27	0.78	0.53	1.14	8	0.62	0.31	1.26	19	0.87	0.55	1.37
					*0.20*				*0.19*				*0.55*		
															
*Waist, adjusted for BMI*															
Quintile 1	n/a	n/a	1963	145	1.00			50	1.00			94	1.00		
Quintile 2	n/a	n/a	1943	165	1.19	0.95	1.49	50	1.10	0.74	1.62	114	1.25	0.95	1.64
Quintile 3	n/a	n/a	1963	146	1.01	0.80	1.27	49	1.01	0.68	1.50	97	1.02	0.77	1.35
Quintile 4	n/a	n/a	1930	178	1.26	1.01	1.57	65	1.37	0.95	1.98	111	1.20	0.92	1.59
Quintile 5	n/a	n/a	1925	183	1.21	0.97	1.51	67	1.28	0.89	1.85	114	1.17	0.89	1.54
					*0.078*				*0.083*				*0.37*		
															
*WHR, adjusted for BMI*															
Quintile 1	n/a	n/a	1937	169	1.00			57	1.00			112	1.00		
Quintile 2	n/a	n/a	1968	139	0.89	0.71	1.12	48	0.95	0.65	1.39	90	0.85	0.64	1.12
Quintile 3	n/a	n/a	1926	181	1.20	0.97	1.49	65	1.36	0.95	1.95	115	1.12	0.86	1.46
Quintile 4	n/a	n/a	1940	166	1.11	0.90	1.38	56	1.18	0.82	1.71	109	1.07	0.82	1.39
Quintile 5	n/a	n/a	1945	162	1.15	0.93	1.43	55	1.25	0.86	1.81	104	1.07	0.82	1.40
					*0.049*				*0.12*				*0.24*		

BMI=body mass index; WHR=waist–hip ratio; HR=hazard ratios.

*P*-values in italics.

a All *P*-values are for linear trends (categorical variables), except where noted.

b Numbers do not always add to 10 564 owing to missing values in some categories.

c Linear *P*-value=0.92.

d Linear *P*-value=0.49.

e Linear *P*-value=0.55.

**Table 2:** Age-adjusted and multivariate^a^ hazard ratios (with lower and upper limits of 95% confidence intervals) for measures of height, obesity, and diabetes prevalence and cases of prostate cancer occurring before age 65^b^

	**Age-adjusted analysis (*n*=8194)^c^**					**Multivariate analysis (*n*=8103)** d		
	**Non-cases (*n*=7992)**	**Incident cases (*n*=202)**						
	***N***	***N***	**HR**	**Lower**	**Upper**	**HR**	**Lower**	**Upper**
*Height*								
Quintile 1	1308	32	1.00			1.00		
Quintile 2	1596	31	0.77	0.47	1.27	0.75	0.46	1.23
Quintile 3	1841	50	1.06	0.68	1.65	1.00	0.64	1.58
Quintile 4	1330	22	0.62	0.36	1.07	0.59	0.34	1.02
Quintile 5	1903	67	1.30	0.85	1.98	1.22	0.79	1.89
			*0.17*			*0.28*		
								
*BMI*								
Underweight	36	3	3.81	1.20	12.08	4.38	1.35	14.17
Normal	3019	78	1.00			1.00		
Overweight	3896	94	0.97	0.72	1.31	0.95	0.70	1.29
Obese	1027	27	1.09	0.71	1.69	1.09	0.70	1.71
*Non-linear P*			*0.13* e			*0.081* f		
								
*BMI*								
Quintile 1	1640	46	1.00			1.00		
Quintile 2	1608	40	0.89	0.58	1.36	0.84	0.55	1.29
Quintile 3	1572	32	0.75	0.48	1.17	0.71	0.45	1.11
Quintile 4	1569	46	1.11	0.74	1.67	1.05	0.69	1.60
Quintile 5	1589	38	0.91	0.59	1.40	0.88	0.56	1.36
			*0.96*			*0.93*		
								
*Body fat %*								
Quintile 1	1603	36	1.00			1.00		
Quintile 2	1597	44	1.20	0.78	1.87	1.20	0.77	1.86
Quintile 3	1607	45	1.27	0.82	1.97	1.28	0.82	1.99
Quintile 4	1558	36	1.08	0.68	1.72	1.05	0.66	1.68
Quintile 5	1576	39	1.19	0.75	1.87	1.20	0.77	1.86
			*0.65*			*0.69*		
								
*Waist*								
Quintile 1	1626	40	1.00			1.00		
Quintile 2	1613	33	0.89	0.56	1.41	0.85	0.53	1.35
Quintile 3	1641	45	1.15	0.75	1.75	1.09	0.71	1.69
Quintile 4	1553	41	1.17	0.76	1.82	1.11	0.71	1.73
Quintile 5	1540	43	1.29	0.84	1.99	1.24	0.79	1.95
			*0.12*			*0.19*		
								
*WHR*								
Quintile 1	1543	34	1.00			1.00		
Quintile 2	1587	30	0.87	0.53	1.42	0.85	0.52	1.40
Quintile 3	1607	39	1.12	0.71	1.78	1.13	0.71	1.80
Quintile 4	1580	52	1.52	0.98	2.34	1.56	1.01	2.43
Quintile 5	1655	47	1.34	0.86	2.09	1.40	0.89	2.21
			*0.023*			*0.012*		
								
*Diabetes?*								
No	1523	198	1.00			1.00		
Yes	6279	4	0.66	0.24	1.77	0.65	0.24	1.75
			*0.41*			*0.39*		
								
*Waist, adjusted for BMI*								
Quintile 1	1562	35	1.00			1.00		
Quintile 2	1626	39	1.05	0.67	1.66	1.05	0.66	1.67
Quintile 3	1625	36	1.04	0.65	1.65	1.03	0.64	1.66
Quintile 4	1609	44	1.28	0.82	2.00	1.29	0.81	2.07
Quintile 5	1551	48	1.57	1.01	2.43	1.59	0.98	2.58
			*0.025*			*0.038*		
								
*WHR, adjusted for BMI*								
Quintile 1	1472	26	1.00			1.00		
Quintile 2	1594	28	0.96	0.56	1.64	0.98	0.57	1.67
Quintile 3	1612	51	1.75	1.09	2.81	1.82	1.13	2.92
Quintile 4	1627	52	1.78	1.11	2.85	1.85	1.15	2.98
Quintile 5	1662	45	1.50	0.93	2.44	1.64	1.00	2.68
			*0.011*			*0.004*		

BMI=body mass index; WHR=waist–hip ratio; HR=hazard ratios.

*P*-values in italics.

a Adjusted for age, height, co-habitation status, socioeconomic status, alcohol habits, smoking habits, prevalent diabetes, total physical activity, birth country, and total intake of EPA, DHA, red meat, and calcium. Height and prevalent diabetes were further adjusted for BMI category.

b All *P*-values are for linear trends (categorical variables), except where noted.

c Numbers do not always add to 8194 owing to missing values in some categories.

d Some persons excluded from the analyses because of missing values, which also affect the number of cases (*n*=200).

e Linear *P*-value=0.86.

f Linear *P*=0.80.

**Table 3:** Multivariate adjusted hazard ratios^a^ (with lower and upper limits of 95% confidence intervals) for measures of height, obesity, and diabetes prevalence and total, aggressive, and non-aggressive cases of prostate cancer^b^

	**Incident prostate cancer, entire cohort (*n*=10 434)** c								
	**All cases (*n*=809)**			**Aggressive cases (*n*=278)**			**Non-aggressive cases (*n*=525)**		
	**HR**	**Lower**	**Upper**	**HR**	**Lower**	**Upper**	**HR**	**Lower**	**Upper**
*Height*									
Quintile 1	1.00			1.00			1.00		
Quintile 2	1.18	0.95	1.47	1.38	0.96	1.98	1.08	0.82	1.43
Quintile 3	1.08	0.86	1.34	1.14	0.79	1.66	1.04	0.79	1.37
Quintile 4	1.04	0.81	1.34	1.01	0.65	1.56	1.08	0.79	1.46
Quintile 5	1.31	1.05	1.64	1.29	0.87	1.91	1.33	1.01	1.76
	*0.081*			*0.64*			*0.055*		
									
*BMI*									
Underweight	2.29	1.13	4.63	3.15	1.15	8.62	0.84	0.63	1.11
Normal	1.00			1.00			1.00		
Overweight	1.02	0.88	1.19	0.99	0.76	1.29	1.16	0.89	1.50
Obese	1.06	0.84	1.33	1.02	0.69	1.52	1.11	0.85	1.44
*Non-linear P*	*0.15* d			*0.16* e			*0.65* f		
									
*BMI*									
Quintile 1	1.00			1.00			1.00		
Quintile 2	0.94	0.76	1.17	0.83	0.57	1.20	1.01	0.76	1.33
Quintile 3	0.96	0.77	1.20	0.89	0.62	1.29	0.99	0.75	1.31
Quintile 4	0.99	0.80	1.24	0.89	0.61	1.28	1.06	0.81	1.40
Quintile 5	0.90	0.72	1.13	0.83	0.57	1.22	0.94	0.71	1.26
	*0.58*			*0.49*			*0.86*		
									
*Body fat %*									
Quintile 1	1.00			1.00			1.00		
Quintile 2	1.22	0.98	1.52	1.33	0.94	1.91	1.13	0.85	1.49
Quintile 3	1.03	0.82	1.30	0.99	0.67	1.45	1.05	0.79	1.39
Quintile 4	1.06	0.84	1.32	0.83	0.56	1.23	1.19	0.90	1.56
Quintile 5	1.10	0.87	1.38	1.05	0.71	1.55	1.12	0.84	1.49
	*0.94*			*0.35*			*0.38*		
									
*Waist*									
Quintile 1	1.00			1.00			1.00		
Quintile 2	0.90	0.71	1.13	0.80	0.55	1.18	0.94	0.70	1.25
Quintile 3	1.05	0.84	1.30	0.93	0.64	1.34	1.10	0.83	1.44
Quintile 4	1.04	0.83	1.29	0.89	0.61	1.30	1.12	0.84	1.47
Quintile 5	1.00	0.80	1.26	0.99	0.68	1.45	1.00	0.75	1.34
	*0.55*			*0.84*			*0.58*		
									
*WHR*									
Quintile 1	1.00			1.00			1.00		
Quintile 2	0.99	0.79	1.23	0.94	0.65	1.37	1.00	0.76	1.31
Quintile 3	0.96	0.77	1.21	1.03	0.71	1.49	0.93	0.70	1.23
Quintile 4	1.23	0.99	1.52	1.27	0.89	1.82	1.21	0.92	1.58
Quintile 5	1.12	0.89	1.41	0.99	0.66	1.47	1.16	0.88	1.53
	*0.073*			*0.46*			*0.12*		
									
*Diabetes?*									
No	1.00			1.00			1.00		
Yes	0.76	0.51	1.11	0.61	0.30	1.25	0.84	0.53	1.33
	*0.15*			*0.18*			*0.45*		
									
*Waist, adjusted for BMI*									
Quintile 1	1.00			1.00			1.00		
Quintile 2	1.17	0.93	1.47	1.11	0.75	1.64	1.21	0.92	1.60
Quintile 3	1.00	0.79	1.27	1.04	0.69	1.55	1.00	0.75	1.33
Quintile 4	1.23	0.98	1.55	1.40	0.95	2.06	1.16	0.87	1.54
Quintile 5	1.17	0.92	1.49	1.29	0.86	1.93	1.11	0.82	1.50
	*0.18*			*0.11*			*0.67*		
									
*WHR, adjusted for BMI*									
Quintile 1	1.00			1.00			1.00		
Quintile 2	0.89	0.71	1.11	0.96	0.65	1.40	0.84	0.63	1.11
Quintile 3	1.22	0.99	1.51	1.34	0.94	1.93	1.16	0.89	1.50
Quintile 4	1.14	0.92	1.42	1.21	0.83	1.75	1.11	0.85	1.44
Quintile 5	1.22	0.98	1.52	1.27	0.87	1.86	1.17	0.89	1.53
	*0.012*			*0.10*			*0.072*		

BMI=body mass index; WHR=waist–hip ratio; HR=hazard ratios.

*P*-values are in italics.

a Adjusted for age, height, co-habitation status, socioeconomic status, alcohol habits, smoking habits, prevalent diabetes, total physical activity, birth country, and total intake of EPA, DHA, red meat, and calcium. Height and prevalent diabetes were further adjusted for BMI category.

b All *P*-values are for linear trends (categorical variables), except where noted.

c Some persons excluded from the analyses because of missing values, which also affect the number of cases.

d Linear *P*=0.95.

e Linear *P*=0.72.

f Linear *P*=0.95.
